# Adverse Childhood Experiences and Risk of Binge Drinking and Drunkenness in Middle-Aged Finnish Men

**DOI:** 10.4061/2011/478741

**Published:** 2011-11-03

**Authors:** Laura Kauhanen, Janne Leino, Hanna-Maaria Lakka, John W. Lynch, Jussi Kauhanen

**Affiliations:** ^1^Department of Public Health, Institute of Public Health and Clinical Nutrition, University of Eastern Finland, Kuopio Campus, P.O. Box 1627, 70211 Kuopio, Finland; ^2^National Institute for Health and Welfare, P.O. Box 30, 00271 Helsinki, Finland; ^3^Discipline of Public Health, School of Population Health and Clinical Practice, University of Adelaide, MDP DX 650 550, Adelaide, SA 5005, Australia; ^4^School of Social and Community Medicine, University of Bristol, Bristol BS8 2PS, UK

## Abstract

*Objective*. The purpose of this study was to investigate associations between adverse childhood experiences and binge drinking and drunkenness in adulthood using both historical and recalled data from childhood. *Methods*. Data on childhood adverse experiences were collected from school health records and questionnaires completed in adulthood. Adulthood data were obtained from the baseline examinations of the male participants (*n* = 2682) in the Kuopio Ischaemic Heart Disease Risk Factor Study (KIHD) in 1984–1989 from eastern Finland. School health records from the 1930s to 1950s were available for a subsample of KIHD men (*n* = 952). *Results*. According to the school health records, men who had adverse childhood experiences had a 1.51-fold (95% CI 1.05 to 2.18) age- and examination-year adjusted odds of binge drinking in adulthood. After adjustment for socioeconomic position in adulthood or behavioural factors in adulthood, the association remained unchanged. Adjustment for socioeconomic position in childhood attenuated these effects. Also the recalled data showed associations with adverse childhood experiences and binge drinking with different beverages. *Conclusions*. Our findings suggest that childhood adversities are associated with increased risk of binge drinking in adulthood.

## 1. Introduction

Binge drinking is commonly defined as consuming five or more servings of alcohol at a time. In Nordic countries, however, consuming six or more alcoholic units of one type of beverage on one drinking occasion is often used in research [1, 2]. It is also a threshold for risky drinking according to the National Institute of Health and Welfare in Finland [3]. Binge drinking is associated with multiple adverse health outcomes [4–9] and is common in the Eastern European and Nordic countries [10, 11]. For example in a study by Paljärvi et al., an increasing volume of alcohol consumption increased the risk of fatal injury [12]. In Finland, alcohol consumption increased quite steadily in the past decades at least until the mid-2000s. In 2008, the total annual consumption per capita was 10.4 litres of pure alcohol, which is somewhat above the average consumption level in the European countries [13]. Approximately, 25% of men and 10% of women binge when they consume alcohol [14]. Binge-drinking behaviour among young people has also been increasing since 2007, except in the youngest age group of 14 years according to the Adolescent Health and Lifestyle survey 2009. In 2009, 22% of boys and 21% of girls aged 14–18 years got heavily drunk on a monthly basis. The prevalence of problem drinkers among young people is 5–10% [15].

The recent trend in alcohol consumption is strongly related to cultural and social processes. Alcohol has become more available in Finland and tracks with the economic growth in the country. Also the drinking culture has become more open, and the settings in which alcohol is consumed have been increasing. When Finland joined European Union in 1995, it was thought that Finnish people would adapt to the Mediterranean drinking habits including drinking wine with the meals. The adaptation process has been somewhat slow, because bingeing behaviour is still common regardless of the beverage type consumed. Also there was an increase in alcohol use and alcohol-related mortality after 2004, when the alcohol tax was lowered [16]. According to Statistics Finland, in 2008 alcohol-related causes were the main cause of death for working-age men and women in Finland [13]. Children and young people are also suffering because of alcohol. The number of children living in the out-of-home custody child care has been increasing in recent years [17]. The main reasons for this are adversities in the household, like mental and substance abuse problems, violence, and poor parenting [18].

Adverse childhood experiences refer to multiple categories of childhood trauma in the household prior to age 18: recurrent physical, emotional, or sexual abuse, an alcohol and/or drug abuser in the household, an incarcerated, chronically depressed, mentally ill, institutionalized, or suicidal household member, mother is treated violently, one or no parents, and emotional or physical neglect [19]. Adverse childhood experiences may lead to different social, emotional and cognitive problems, and lower socioeconomic achievement in childhood and adulthood, which in turn may lead to adoption of risky lifestyle behaviors and premature death [19]. Particular adverse childhood experiences may initiate early alcohol intake and drinking to cope with problems rather than to be social or for pleasure [20]. For example, parents' divorce and poor quality of family relationships (inadequate parenting, parents' problem drinking) have been shown to predict early drinking and alcohol abuse in the offspring [20–23]. Moreover, studies on alcohol use among adolescents have reported an association between domestic violence, discipline, peer group, and psychosocial problem with early alcohol intake [19, 22, 24, 25]. However, Yang et al. did not find an association between negative life events during childhood and binge drinking in adulthood using the Kuopio Ischaemic Heart Diseases Risk Factor (KIHD) questionnaire-based data. They studied the influence of death in the family, illness of the parents, divorce, and separation from the parents due to war in their index of early life negative experiences [26]. Many of the studies on adverse childhood experiences have used recalled information from childhood that can underestimate the effect of childhood adversities [19, 24–27].

The purpose of this study was to examine the role of adverse childhood experiences using two sources of information—one collected in childhood by a nurse or a doctor who made home visits and saw children in school, and the other collected via adult recall of childhood adversities, as predictors of binge drinking and drunkenness in adulthood. Information on adverse childhood experiences is less likely to suffer from recall bias, which is the biggest threat to validity, when it is measured by a more objective report of a nurse or doctor in childhood, instead of asking adults to recall experiences of adversity in childhood. Additionally, we examined the mediating role of behavioural factors and socioeconomic position both in childhood and adulthood on the association between adverse childhood experiences and binge drinking in adulthood.

## 2. Materials and Methods

### 2.1. Study Population

The subjects were participants in the Kuopio Ischemic Heart Disease Risk Factor study (KIHD) which is a prospective population-based study designed to investigate risk factors for cardiovascular diseases, including psychosocial and socioeconomic factors, in middle-aged men from Eastern Finland. The original study population consisted of a random age-stratified sample of 2682 men who were 42, 48, 54, or 60 years of age at baseline in 1984. The Research Ethics Committee of the University of Kuopio approved the study. The school health records were available for 952 (35.5%) men. There were missing data because some of the archives, where school health records were stored, were destroyed during fires and World War II. The historical final sample was 839 after men who had not consumed alcohol during the past year were excluded from the analysis (*n* = 366). The questionnaire-based adverse childhood experiences variable was available for 2682 (100.0%) men. The final sample for the questionnaire-based analysis was 2311 after men who had not consumed alcohol during the past year were excluded from the analysis (*n* = 366). A comparison of the historical study sample with the rest of the KIHD cohort revealed that men in the subsample were on average somewhat younger, their education, occupational, and income levels were higher, they were physically more active and they have smoked cigarettes less than the rest of the KIHD cohort (Table 1).

### 2.2. Adverse Childhood Experiences

Adverse childhood experiences were measured in two ways: (1) historical data from school health records and (2) questionnaire-based recall information. 

(1) Childhood information was obtained from school health records completed by the school health nurses and doctors in the 1930s to 1950s and maintained by the schools or the municipality archives. The intention of the school health records was to have nurses and doctors collect data on health status, school attendance, behaviour at school, general parenting practices and hygiene/cleanliness of the child, and socioeconomic circumstances of all children they visited at home based. This program was a universal service in Finland from the late 1920s. 

Adverse childhood experiences were defined as present if a school health nurse had reported one or more of the following:

   (1) father's alcohol problem;
   (2) relative's alcohol problem;
   (3) parents' divorce;
   (4) mother's death;
   (5) father's death;
   (6) death of a sibling.

These items were analysed as a summary variable to represent the total adverse childhood experiences score. 

(2) Questionnaire-based adverse childhood experiences recalled in adulthood were defined from the questions (Appendix A) relating to

   (1) father's alcohol problem;
   (2) mother's alcohol problem;
   (3) father's death;
   (4) mother's death;
   (5) parents' divorce;
   (6) father was stern/punishing;
   (7) mother was stern/punishing;
   (8) quarrelsome home;
   (9) unhappy and difficult childhood.

These items were scored dichotomously. Items (1) and (2) were summed as “parents alcohol problem,” items (3)-(4) were summed as “parental death,” item (5) was analysed separately as “parents' divorce,” items (6) and (7) were summed as “poor parenting,” item (8) was analysed separately as “quarrelsome home” and item (9) was analysed separately as “unhappy childhood.” In addition, all items were summed to obtain the adverse childhood experiences score with four categories (0, 1, 2, or ≥3).

### 2.3. Baseline Covariates

#### 2.3.1. Age and Examination Year

Age was categorized into four groups: 42, 48, 54 years, and 60 years. Examination year was categorized from 1984 to 1989.

#### 2.3.2. Behavioural Factors

Alcohol consumption in grams per week was assessed with a structured quantity and frequency method using the Nordic alcohol consumption inventory [4]. Usual frequency of intake and usual dose (in glasses or bottles) were recorded for each type of drink (beer, wine, fortified wine, spirits) with a structured response form. We calculated the measure for average weekly intake of various drinks on the basis of known alcohol content. One bottle (0,33 L) of beer, a glass (12 cL) of wine, and one shot (4 cL) of strong spirits contains 12 g of ethanol. One bottle of wine (75 cL) contains 72 grams of ethanol. One bottle (75 cL) of fortified wine contains 120/132 grams of ethanol. The usual dose of beer was determined according to the following scale: <1 bottle, 1 bottle, <2 bottles, 2 bottles, 3 bottles, 4 or 5 bottles, 6–9 bottles, and >9 bottles. We used the total mean consumption of all alcoholic drinks (grams of ethanol/week) as the covariate in adjusted models. Leisure-time physical activity in hours per year from a 12-month history has been described previously [28]. Cigarette smoking was estimated by self-reporting and converted to pack-years (the average number of cigarettes per day times the number of years smoked). Body mass index was calculated as the ratio of weight to the square of height in metres (kg/m^2^) [29].

#### 2.3.3. Childhood Socioeconomic Variables

Socioeconomic position in childhood was a summary variable including education of father and mother, occupation of father and mother, and the number of rooms at the age of ten. These variables were summed, and individuals were assigned to high, middle, or low childhood socioeconomic position by the index tertiles [26]. Education was also included in the analysis of childhood socioeconomic position. It was categorized into four groups: less than elementary, elementary, full or some secondary, and high school or above.

#### 2.3.4. Adulthood Socioeconomic Variables

Adulthood socioeconomic position was assessed by the self-report of annual personal income and occupation. Occupation was categorized into three groups: 1 = farmer, 2 = blue collar, and 3 = white collar.

### 2.4. Outcomes

#### 2.4.1. Binge Drinking

Usual frequency of intake and quantity of beer, wine, fortified wine, and spirits were measured [4]. Binge drinking was computed from the questions where an average number of drinks consumed at one occasion were asked. The questions were designed for each beverage specifically and phrased: “How much of  “*⋯*” did you drink at one occasion?” The question had 8 quantity categories formed to the specific beverage. The quantities referred to the number of glassfuls (wines), bottles (beer, wine, hard liquor), and restaurant portions (hard liquor) taking into account the alcohol content of each serving size [3, 4]. Binge drinking was defined as any of the following: drinking 6 or more bottles of beer, 51 centilitres (cL) or more wine, 38 cL or more of fortified wine, and 31 cL or more of spirits per occasion during the past 12 months. In addition, a summary variable was constructed to represent bingeing with any beverage. There were 527 binge drinkers, including 79 men who reported bingeing on beer, 76 on wine, 147 on fortified wine, and 424 on hard liquor. Binge drinking was also assessed by self-report of being drunk during the last 12 months. According to the Alcohol in Europe study, Finnish adults get drunk approximately in every 11th day [30]. Being-drunk variable was dichotomised, and the cutoff point was having been drunk once a week or more often. There were 181 men who had been drunk once a week or more often.

### 2.5. Statistical Analysis

Chi-squared tests and independent samples *T*-tests were used to assess differences in baseline characteristics between the groups. The association between adverse childhood experiences and the risk of binge drinking and drunkenness in later life was analysed with logistic regression models. To be at risk of binge drinking, a subject had to consume alcohol. Thus, we excluded the men who had not consumed alcohol during the past year from the analysis. Two separate set of analyses were performed (1) with historical and (2) with questionnaire-based recall childhood data. 

(1) There were 839 cases in the historical analysis. Father had died from 102 men, mother had died from 18 men, and a sibling had died from 76 men. There were 9 men whose parents' had divorced, 16 men whose fathers had an alcohol problem, and 14 men whose mothers had an alcohol problem. These items were analysed as a summary variable to represent the total adverse childhood experiences score. If there was no mention of the items (1)–(6), the man was defined as not having adverse childhood experiences. Men with adverse childhood experiences formed the index group (24.7%) and men without adverse childhood experiences were a reference group (75.3%) in the analysis. 

(2) In the questionnaire-based childhood analysis there were 2311 cases. There were 179 men whose fathers had an alcohol problem, and 17 men whose mothers had an alcohol problem. A father had died from 240 men, and a mother had died from 99 men. Parents' had divorced from 40 men. There were 314 men who had stern/punishing fathers and 147 men who had stern/punishing mothers. There were 182 men who had a quarrelsome home, and 163 men who had an unhappy and difficult childhood. Parents' alcohol problem was recorded for 8.5% of men, parental death was recorded for 14.0% of men, parents' divorce was recorded for 1.7% of men, poor parenting was recorded for 18.8% of men, quarrelsome home was recorded for 7.9% of men, and unhappy childhood was recorded for 7.1% of men. In the analysis of adverse childhood experiences, summary score dummy-variables were created to represent four categories of adversities (0, 1, 2, or ≥3). There were 303 men without adverse childhood experiences, 1157 men with one adverse experience, 578 men with two adverse experiences, and 273 men with three or more adverse experiences. All dummy variables were entered to the model, except the 0 category, which was regarded as the reference group. In addition, we tested linear trend of different categories of ACEs in regard binge drinking and drunkenness with polynomial contrast in logistic regression models. All analyses were performed using SPSS for Windows 17.0. Covariates were entered uncategorized into the models with the exception of age (group), examination year, educational and occupational levels, and childhood socioeconomic position. 

A sequence of models was examined to analyse the relation between adverse childhood experiences and binge-drinking behaviour. Model 1 included age- and examination year. Childhood socioeconomic position and education were added in Model 2. Model 3 was the same as Model 1 and additionally adjusted for adulthood socioeconomic position (income, occupation). Model 4 was the same as Model 1 and additionally adjusted for behavioural characteristics in adulthood (average alcohol consumption, smoking, body mass index, physical activity). Model 5 consisted of all covariates.

## 3. Results

### 3.1. Historical Childhood Data

Table 2 shows the mean ± standard deviation or prevalence for the baseline covariates: age, the behavioural factors, education, occupation, childhood socioeconomic position, and income for men with and without adverse childhood experiences. Childhood socioeconomic position was lower in men with adverse childhood experiences compared to those men without these experiences. In addition, they had smoked cigarettes more than men without adverse experiences in childhood. There were no differences between groups in other covariates. 

Table 3 shows that men with adverse childhood experiences had a 1.51-fold (95% confidence interval (CI) 1.05 to 2.18) age- and examination-year adjusted odds of binge drinking with any beverage (spirits, beer, wine, strong wine). The association remained unchanged after adjustment for socioeconomic position in adulthood and behavioural factors in adulthood (Models 3 and 4), but adjustments for socioeconomic position in childhood (Model 2) and all covariates (Model 5) attenuated the effect to nonsignificance. There was also an association with adverse childhood experiences and bingeing with spirits and being drunk once a week or more often over the last 12 months when adjusted for age, examination year, and behavioural factors in adulthood (Model 4). Adjustments with the socioeconomic position in childhood or adulthood attenuated these effects.

### 3.2. Questionnaire-Based Childhood Data

Table 2 shows the mean ± standard deviation or prevalence for the baseline covariates: age, the behavioural factors, education, occupation, childhood socioeconomic position, and income for men with and without adverse childhood experiences. Educational level, occupational level, income level, and childhood socioeconomic position were lower in men with adverse childhood experiences compared to those men without these experiences. They were also older than men without adverse childhood experiences. There were no differences between groups in other covariates. 

Table 4 shows that men who reported parental alcohol problem had a 1.49-fold (95% CI 1.05 to 2.02) age- and examination-year adjusted odds of binge drinking with spirits, 2.23-fold (1.20 to 4.16) odds of binge drinking with beer, 2.46-fold (1.29 to 4.69) odds of binge drinking with wine, and 1.54-fold (1.11 to 2.13) odds of bingeing with any beverage (spirits, beer, wine, fortified wine). The associations remained unchanged for bingeing with beer and wine after adjusting for socioeconomic and behavioural factors. After controlling for behavioural factors in adulthood or all covariates, binge drinking with spirits and any beverage were not reaching statistical significance anymore. Parental death or divorce showed no associations with binge drinking in adulthood. Poor parenting, quarrelsome home, and unhappy childhood showed associations with bingeing with spirits and overall binge drinking. The men who had three or more childhood adversities had greater odds of binge-drinking behaviour compared to men without, or with one or two adversities. According to the trend test there were statistically significant results with adverse childhood experiences score and binge drinking with different beverages, except in regard to binge drinking with strong wine in models 2, 4, and 5 and with beer in models 4 and 5. In the recall data there were no associations between adverse childhood experiences and being drunk once a week or more often, except parents' divorce showed a twofold odds of being drunk, but the results were not statistically significant.

## 4. Discussion

Our findings suggest that certain adverse childhood experiences increase the risk of binge drinking in adulthood even after adjustment for behavioural factors in adulthood and for the socioeconomic position in adulthood. The association was seen in both historical and the questionnaire-based data providing some cross-validation of the effects observed. Authentic historical records may give additional and more accurate information of the association although in some of the analyses there is the effect of power loss due to smaller numbers available for the analyses. According to the historical records, bingeing with any beverage showed the strongest associations with adverse childhood experiences after adjusting for age, examination year, socioeconomic position in adulthood, and behavioural factors. After adjusting for socioeconomic position in childhood or all covariates, the effect estimates did not change much, but the results no longer reached traditional levels of statistical significance. 

Using questionnaire-based recall information the effect of parents' alcohol problems, death, divorce, poor parenting style, quarrelsome home, and unhappy childhood were examined separately. Parents' alcohol problems, a punishing parenting style, quarrelsome home, and unhappy childhood were associated with higher odds of binge-drinking behaviour. There is some evidence that adverse childhood experiences are interrelated [19]. For example, parental substance use may increase the risk of inconsistent parenting, like harsh parental discipline and lack of warmth and nurturance. This result corresponds to previous research suggesting that punishing parenting style increases the risk of early alcohol drinking in adolescence [22]. Genetics may also explain a part of the observed association, as alcohol dependence is partly inherited [31]. On the other hand, it has been found that foster parents' alcohol problems have an influence on the drinking behaviour of the adopted children [32]. 

The death of a parent is regarded as one of the most stressful life events that a child can experience [33]. In a study by Melhem et al., sudden parental death was associated with higher rates of personality and substance use disorders among the offspring [34]. However, Muñiz-Cohen et al. did not find an increased risk of health risk behaviours among the bereaved youth after nine months of the parental death by suicide, accident or a sudden natural death [35]. In our study parental death, or divorce did not show any effect on binge drinking with different beverages in adulthood. However, there was an increased odds of being drunk once or more often a week in men whose parents' had divorced, but the results were not statistically significant. This result gives some support to the previous studies; for example, Kendler et al. found that parental separation due to divorce and other reasons than death increased the risk of alcohol dependence [21]. Also Huurre et al. found that parental divorce predicted an excessive alcohol use in adulthood [36]. It is suggested that the association between parental loss and alcoholism occurs because of both the environmental effects of parental loss and the genetic transmission of alcoholism risk [21]. Parental loss may also lead to the path of socioeconomic disparities in health. For example, in our previous study social disadvantage in childhood was associated with an increased risk of acute coronary events in adulthood [37]. However, Yang et al. did not find an association with negative childhood experiences and binge drinking using the same KIHD data; they examined the effect of separation from the parents and parental illness but did not include parents' alcohol problems, poor parenting style, or quarrelsome home in their index. In addition, they did not have historical data in their analysis [26]. We examined also the cumulative effect of adverse childhood experiences. The men who had had three or more childhood adversities had greater odds of binge drinking behaviour compared with men without, or with one or two adversities, suggesting a dose-response relationship between the adversities in childhood and binge drinking in adulthood.

In the retrospective study design recall bias can cause underestimation of the true impact of childhood circumstances, as people may not remember all the details of the past. For example, the inability to remember childhood events is suggested to be associated with adverse childhood experiences, such as childhood sexual abuse [38]. According to Hardt and Rutter retrospective reports in adulthood of adverse experiences in childhood can have a high rate of false negatives but few false positive reports [39]. In the present study, the school health nurses and doctors were sent to observe the home circumstances of the boys and they regularly followed up the health and behaviour of the children. The measure for adverse childhood experiences is quite broad, but its advantage is that the measure comes from the original observations by health professionals. Overall, the results from the historical analyses could be regarded as more objective and therefore more reliable than the results from the recalled information on childhood adversities although some of the results in the historical analyses were not precise enough to draw a reliable conclusion due to the small sample size. The findings from historical analyses might have been significant in a larger sample.

By the standards of modern epidemiologic research, the school health records are a very old source of information. The records were stored by either individual schools or municipalities. Many of the old schools have been closed since those days, and at least one municipal archive is known to have been destroyed in a fire. About 9% of the original KIHD sample were Karelian refugees, who had to leave behind their schools, and in most cases, their health records, in the course of World War II. However, there is no indication that the historical final sample (*n* = 839) is in any way gravely misrepresentative of the total KIHD study population although the historical sample study participants were somewhat younger, had higher socioeconomic position, and were less likely to smoke cigarettes. A potential limitation of the study is that it included only men, so the results may not be generalizable to women or outside the Finnish population. Another limitation is that although the use of external raters may be more objective than self-report for childhood factors, it is not possible to know what instructions were given to the nurses and doctors about how to report what they observed or how diligently they carried out this task because this data was not collected as part of a research study. In addition, underreporting of alcohol consumption would result in the odds ratios being biased toward the null hypothesis. Furthermore, the selection of nondrinkers as a reference group has been questioned because this group may include exdrinkers who had to stop drinking because of health problems [40]. In this study many of those who do not drink do so because they have been heavy drinkers before and may have quit for medical reasons, so including them in the “nondrinkers” will bias associations to the null. That is why abstainers were excluded from the analyses. 

This study provides evidence that childhood adversities may predict adverse drinking habits in adulthood that can have implications for social, emotional, and physical dimensions of health. It would be useful in future research to have more information about coexisting childhood stressors within families and societies at large to understand the process of binge-drinking behaviour and its association with human health risks.

## 5. Conclusions

Both historical archive data and the recall data suggest that adverse childhood experiences are associated with increased odds of binge-drinking behaviour in adulthood. Authentic historical records may give additional and more accurate information of the association, although in some of the analyses there is the effect of power loss due to smaller numbers in the analyses.

## Figures and Tables

**Table 1:** Comparison of the historical study sample (*n* = 839) with the rest of the Kuopio Ischaemic Risk Factor study (KIHD) cohort (*n* = 1472).

Covariates	Mean (SD) or proportion (%)		
	Historical study sample (*n* = 839)	The rest of the KIHD cohort (*n* = 1472)	*P* values for the difference between groups
Age group (%)			
(1) (42 years)	24.7	6.7	<0.001
(2) (48 years)	19.9	10.0	
(3) (54 years)	44.8	66.4	
(4) (60 years)	10.6	16.9	
Educational level (%)			
(1) less than elementary	7.5	11.1	<0.001
(2) elementary	42.6	49.5	
(3) full or some secondary	42.8	31.9	
(4) high school or above	7.2	7.4	
Occupational level (%)			
(1) farmer	11.7	17.8	<0.001
(2) blue collar	45.8	42.1	
(3) white collar	42.6	40.2	
Smoking history (pack/years)	163.7 (299.6)	200.7 (377.2)	0.011
BMI (kg/m^2^)	26.8 (3.6)	26.9 (3.5)	0.604
LDL cholesterol (mmol/L)	4.0 (1.0)	4.1 (1.0)	0.008
HDL cholesterol (mmol/L)	1.3 (0.3)	1.3 (0.3)	0.424
SBP (mm Hg)	133.5 (16.3)	134.5 (17.1)	0.198
Leisure time physical activity (h/year)	123.5 (153.8)	106.7 (129.7)	0.008
Alcohol consumption (g/week)	92.7 (151.7)	85.9 (139.2)	0.281
Income (marks/year)	83,983.2 (54174.6)	77,512.3 (52398.8)	0.005

BMI: body mass index; LDL: low density lipoprotein; HDL: high density lipoprotein; SBP: systolic blood pressure.

**Table 2:** Baseline characteristics of men with and without adverse childhood experiences using historical childhood data (*n* = 839) and questionnaire-based data from adulthood (*n* = 2311) from the Kuopio Ischaemic Heart Disease Risk Factor study baseline examinations in 1984.

Covariates	Mean (SD) or proportion (%)					
	Historical childhood data (*n* = 839)			Questionnaire-based childhood data (*n* = 2311)		
	Men with adverse childhood experiences (*n* = 207)	Men without adverse childhood experiences (*n* = 632)	*P*-values for the difference between groups	Men with adverse childhood experiences (*n* = 2008)	Men without adverse childhood experiences (*n* = 303)	*P*-values for the difference between groups
Age group (%)						
42	18.4	26.7	0.074	12.9	15.2	0.021
48	19.8	19.9		13.2	15.8	
54	48.8	43.5		59.8	50.5	
60	13.0	9.8		14.0	18.5	
Educational level (%)						
(1) less than elementary	10.1	6.7	0.341	10.0	8.4	<0.001
(2) elementary	41.5	42.9		48.9	34.7	
(3) full or some secondary	43.0	42.7		35.2	40.3	
(4) high school or above	5.3	7.8		5.9	16.8	
Occupation (%)						
(1) farmer	10.1	12.2	0.784	15.8	13.5	<0.001
(2) blue collar	46.4	45.6		45.1	32.0	
(3) white collar	43.5	42.7		39.0	54.8	
Childhood SEP (%)						
(1) low	34.8	29.0	0.003	32.8	20.8	<0.001
(2) middle	47.8	41.8		43.7	37.0	
(3) high	17.4	29.3		23.6	42.2	
Smoking history (pack/years)	202.5 (327.9)	151.1 (289.0)	0.048	188.4 (354.9)	180.0 (327.6)	0.703
Body mass index (kg/m^2^)	26.6 (3.5)	26.9 (3.7)	0.294	26.9 (3.6)	26.6 (3.5)	0.127
Leisure time physical activity (h/year)	130.1 (157.3)	121.4 (152.7)	0.484	113.1 (141.5)	110.7 (122.9)	0.784
Alcohol consumption (g/week)	100.5 (219.0)	90.1 (122.0)	0.395	89.9 (149.6)	78.4 (96.6)	0.195
Median	51.1	46.9		44.0	36.7	
Maximum	2853.0	867.9		2853.0	633.0	
Income (marks/year)	80 561.6 (51384.1)	85 096.4 (55045.9)	0.300	776160.0 (50958.9)	94 723.9 (63847.4)	<0.001
Median	70 000.0	76 000.0		69 000.0	81 000.0	
Maximum	360 000.0	550 000.0		550 000.0	550 000.0	

SEP: socioeconomic position.

**Table 3:** Historical childhood data. Odds ratios (OR) of binge-drinking and drunkenness in men with adverse childhood experiences (*n* = 207), compared with men without adverse childhood experiences (*n* = 632) as a reference group in the KIHD study (*n* = 839).

OR (95% CI)						
	Spirits ≥ 31 cL/session	Beer ≥ 6 bottles/session	Wine ≥ 51 cL/session	Fortified wine ≥ 38 cL/session	Bingeing with any beverage/session	Being drunk ≥ 1 times/week
No adverse childhood experiences (reference)	1.0	1.0	1.0	1.0	1.0	1.0
Adverse childhood experience, adjusted for						
Model 1	1.49 (1.00–2.21)	1.77 (0.83–3.79)	1.34 (0.58–3.09)	1.46 (0.76–2.78)	1.51 (1.05–2.18)	1.47 (0.85–2.53)
Model 2	1.35 (0.90–2.02)	1.72 (0.80–3.71)	1.46 (0.62–3.43)	1.27 (0.65–2.49)	1.42 (0.98–2.06)	1.38 (0.80–2.39)
Model 3	1.40 (0.93–2.12)	1.74 (0.81–3.73)	1.38 (0.60–3.17)	1.32 (0.67–2.61)	1.47 (1.01–2.14)	1.43 (0.82–2.47)
Model 4	1.63 (1.01–2.60)	1.82 (0.79–4.20)	1.69 (0.65–4.37)	1.37 (0.62–2.99)	1.58 (1.02–2.45)	2.13 (1.06–4.28)
Model 5	1.53 (0.93–2.52)	1.79 (0.76–4.21)	1.73 (0.65–4.62)	1.37 (0.59–3.17)	1.53 (0.96–2.43)	1.94 (0.95–3.98)

Model 1: adjusted for age, examination year.

Model 2: the same as model 1 and childhood socioeconomic position, education.

Model 3: the same as model 1 and adulthood socioeconomic position (income level, occupation).

Model 4: the same as model 1 and behavioural factors in adulthood (body mass index, leisure time physical activity, smoking, alcohol consumption).

Model 5: the same as model 2 and adulthood socioeconomic position and behavioural factors in adulthood.

**Table 4:** Questionnaire-based childhood data. Odds ratios (OR) for the relationship between adverse childhood experiences and binge drinking and drunkenness in the KIHD study (*n* = 2311).

OR (95% CI)						
	Spirits ≥ 31 cL/session	Beer ≥ 6 bottles/session	Wine ≥ 51 cL/session	Fortified wine ≥ 38 cL/session	Bingeing with any beverage/session	Being drunk ≥ 1 times/week
Category of adverse childhood experiences (ACE), adjusted for						
Model 1						
Parents alcohol problem						
No (referent)	1.0	1.0	1.0	1.0	1.0	1.0
Yes	1.49 (1.05–2.12)	2. 23 (1.20–4.16)	2.46 (1.29–4.69)	1.53 (0.87–2.70)	1.54 (1.11–2.13)	1.29 (0.78–2.13)
Parental death						
No (referent)	1.0	1.0	1.0	1.0	1.0	1.0
Yes	1.07 (0.79–1.46)	0.75 (0.35–1.58)	1.12 (0.56–2.23)	1.32 (0.80–2.18)	1.05 (0.80–1.40)	0.80 (0.49–1.30)
Parent's divorce						
No (referent)	1.0	1.0	1.0	1.0	1.0	1.0
Yes	1.10 (0.50–2.42)	0.67 (0.09–4.99)	1.25 (0.29–5.46)	1.07 (0.36–3.15)	1.27 (0.63–2.56)	2.04 (0.84–4.93)
Poor parenting						
No (referent)	1.0	1.0	1.0	1.0	1.0	1.0
Yes	1.46 (1.13–1.88)	1.40 (0.83–2.35)	1.48 (0.86–2.52)	1.04 (0.66–1.64)	1.48 (1.17–1.87)	1.38 (0.96–1.97)
Quarrelsome home						
No (referent)	1.0	1.0	1.0	1.0	1.0	1.0
Yes	1.76 (1.24–2.49)	1.12 (0.50–2.48)	1.98 (0.98–4.02)	1.34 (0.73–2.45)	1.64 (1.18–2.28)	1.19 (0.70–2.01)
Unhappy childhood						
No (referent)	1.0	1.0	1.0	1.0	1.0	1.0
Yes	1.73 (1.21–2.49)	1.47 (0.66–3.28)	1.43 (0.60–3.44)	1.13 (0.56–2.27)	1.65 (1.17–2.33)	1.22 (0.70–2.13)
ACE score						
0 (referent)	1.0	1.0	1.0	1.0	1.0	1.0
1	1.56 (1.06–2.29)	3.24 (1.15–9.11)	1.92 (0.79–4.67)	1.89 (0.97–3.68)	1.56 (1.10–2.20)	1.38 (0.82–2.34)
2	1.79 (1.19–2.68)	2.00 (0.65–6.15)	1.86 (0.72–4.83)	2.47 (1.24–4.92)	1.77 (1.23–2.54)	1.35 (0.76–2.38)
3	2.57 (1.65–3.98)	4.02 (1.30–12.42)	4.35 (1.67–11.31)	2.24 (1.02–4.91)	2.49 (1.68–3.71)	1.81 (0.98–3.36)
	*P* < 0.001*	*P* = 0.030	*P* = 0.002	*P* = 0.020	*P* < 0.001	*P* = 0.068
Model 2						
Parents alcohol problem						
No (referent)	1.0	1.0	1.0	1.0	1.0	1.0
Yes	1.52 (1.06–2.16)	2.21 (1.18–4.11)	2.18 (1.14–4.16)	1.56 (0.87–2.78)	1.57 (1.13–2.19)	1.31 (0.79–2.18)
Parental death						
No (referent)	1.0	1.0	1.0	1.0	1.0	1.0
Yes	1.02 (0.75–1.39)	0.74 (0.35–1.56)	1.13 (0.56–2.26)	1.13 (0.68–1.90)	1.02 (0.78–1.36)	0.78 (0.48–1.26)
Parent's divorce						
No (referent)	1.0	1.0	1.0	1.0	1.0	1.0
Yes	1.28 (0.57–2.85)	0.73 (0.10–5.45)	1.33 (0.30–5.87)	1.21 (0.40–3.71)	1.46 (0.71–3.00)	2.35 (0.96–5.76)
Poor parenting						
No (referent)	1.0	1.0	1.0	1.0	1.0	1.0
Yes	1.46 (1.13–1.89)	1.38 (0.82–2.34)	1.44 (0.84–2.47)	1.03 (0.65–1.66)	1.50 (1.18–1.91)	1.38 (0.65–1.01)
Quarrelsome home						
No (referent)	1.0	1.0	1.0	1.0	1.0	1.0
Yes	1.65 (1.15–2.35)	1.10 (0.49–2.44)	1.88 (0.92–3.86)	1.17 (0.63–2.19)	1.56 (1.11–2.18)	1.13 (0.66–1.91)
Unhappy childhood						
No (referent)	1.0	1.0	1.0	1.0	1.0	1.0
Yes	1.58 (1.09–2.29)	1.48 (0.66–3.31)	1.35 (0.56–3.27)	1.05 (0.51–2.15)	1.53 (1.08–2.18)	1.13 (0.65–1.98)
ACE score						
0 (referent)	1.0	1.0	1.0	1.0	1.0	1.0
1	1.32 (0.89–1.96)	3.13 (1.11–8.83)	1.70 (0.70–4.16)	1.48 (0.75–2.95)	1.35 (0.95–1.92)	1.21 (0.72–2.06)
2	1.44 (0.95–2.20)	1.84 (0.60–5.70)	1.68 (0.64–4.39)	1.81 (0.88–3.72)	1.50 (1.03–2.18)	1.17 (0.66–2.88)
3	2.17 (1.38–3.42)	4.04 (1.30–12.55)	4.14 (1.57–10.95)	1.84 (0.80–4.22)	2.26 (1.50–3.42)	1.56 (0.83–2.91)
	*P* = 0.001	*P* = 0.038	*P* = 0.005	*P* = 0.116	*P* < 0.001	*P* = 0.186
Model 3						
Parents alcohol problem						
No (referent)	1.0	1.0	1.0	1.0	1.0	1.0
Yes	1.52 (1.06–2.18)	2.14 (1.14–4.00)	2.24 (1.17–4.26)	1.45 (0.81–2.60)	1.56 (1.12–2.18)	1.26 (0.76–2.09)
Parental death						
No (referent)	1.0	1.0	1.0	1.0	1.0	1.0
Yes	1.03 (0.76–1.40)	0.72 (0.34–1.51)	1.08 (0.54–2.16)	1.23 (0.74–2.06)	1.03 (0.77–1.37)	0.78 (0.48–1.26)
Parent's divorce						
No (referent)	1.0	1.0	1.0	1.0	1.0	1.0
Yes	1.14 (0.49–2.67)	0.77 (0.10–5.81)	1.39 (0.31–6.13)	1.30 (0.43–4.02)	1.36 (0.64–2.87)	2.33 (0.95–5.72)
Poor parenting						
No (referent)	1.0	1.0	1.0	1.0	1.0	1.0
Yes	1.49 (1.15–1.94)	1.40 (0.83–2.37)	1.46 (0.85–2.51)	1.05 (0.66–1.68)	1.52 (1.20–1.94)	1.40 (0.98–2.01)
Quarrelsome home						
No (referent)	1.0	1.0	1.0	1.0	1.0	1.0
Yes	1.76 (1.23–2.53)	1.10 (0.49–2.45)	1.86 (0.91–3.81)	1.33 (0.72–2.48)	1.65 (1.17–2.32)	1.19 (0.70–2.02)
Unhappy childhood						
No (referent)	1.0	1.0	1.0	1.0	1.0	1.0
Yes	1.67 (1.16–2.42)	1.36 (0.61–3.05)	1.29 (0.54–3.11)	1.02 (0.50–2.08)	1.57 (1.11–2.23)	1.14 (0.65–2.08)
ACE score						
0 (referent)	1.0	1.0	1.0	1.0	1.0	1.0
1	1.35 (0.91–2.00)	3.05 (1.08–8.60)	1.70 (0.70–4.15)	1.81 (0.89–3.67)	1.42 (1.00–2.02)	1.30 (0.77–2.20)
2	1.48 (0.97–2.25)	1.82 (0.59–5.63)	1.73 (0.66–4.50)	2.39 (1.14–5.00)	1.57 (1.08–2.30)	1.28 (0.72–2.26)
3	2.32 (1.48–3.66)	3.80 (1.23–11.79)	3.94 (1.50–10.33)	2.27 (0.98–5.24)	2.43 (1.60–3.67)	1.68 (0.90–3.13)
	*P* < 0.001	*P* = 0.047	*P* = 0.006	*P* = 0.035	*P* < 0.001	*P* = 0.112
Model 4						
Parents alcohol problem						
No (referent)	1.0	1.0	1.0	1.0	1.0	1.0
Yes	1.37 (0.89–2.10)	2.06 (1.04–4.09)	2.30 (1.11–4.77)	1.31 (0.68–2.55)	1.51 (1.00–2.26)	0.88 (0.44–1.75)
Parental death						
No (referent)	1.0	1.0	1.0	1.0	1.0	1.0
Yes	1.09 (0.76–1.57)	0.73 (0.33–1.63)	1.20 (0.56–2.64)	1.28 (0.72–2.29)	1.09 (0.78–1.54)	0.68 (0.35–1.31)
Parent's divorce						
No (referent)	1.0	1.0	1.0	1.0	1.0	1.0
Yes	0.91 (0.32–2.59)	0.85 (0.11–6.68)	0.68 (0.08–5.51)	0.67 (0.15–2.89)	0.97 (0.37–2.51)	2.52 (0.81–7.86)
Poor parenting						
No (referent)	1.0	1.0	1.0	1.0	1.0	1.0
Yes	1.34 (0.98–1.82)	1.27 (0.72–2.26)	1.65 (0.91–2.99)	1.04 (0.62–1.76)	1.46 (1.09–1.94)	1.13 (0.70–1.83)
Quarrelsome home						
No (referent)	1.0	1.0	1.0	1.0	1.0	1.0
Yes	2.17 (1.44–3.27)	1.26 (1.44–3.27)	2.97 (1.39–6.34)	1.93 (0.99–3.75)	1.99 (1.34–2.96)	1.42 (0.73–2.77)
Unhappy childhood						
No (referent)	1.0	1.0	1.0	1.0	1.0	1.0
Yes	2.00 (1.32–3.05)	1.56 (0.66–3.69)	1.55 (0.59–4.06)	0.98 (0.43–2.22)	1.78 (1.18–2.67)	1.42 (0.70–2.86)
ACE score						
0 (referent)	1.0	1.0	1.0	1.0	1.0	1.0
1	1.80 (1.14–2.86)	2.85 (0.98–8.27)	2.84 (0.94–8.60)	1.80 (0.87–3.70)	1.93 (1.26–2.96)	1.18 (0.63–2.20)
2	1.70 (1.04–2.80)	1.62 (0.51–5.21)	2.45 (0.75–7.94)	2.15 (1.00–4.61)	1.87 (1.18–2.96)	0.82 (0.40–1.66)
3	3.43 (2.02–5.83)	3.85 (1.19–12.46)	7.91 (2.42–25.82)	2.31 (0.94–5.67)	3.72 (2.27–6.12)	1.70 (0.80–3.63)
	*P* < 0.001	*P* = 0.062	*P* = 0.001	*P* = 0.062	*P* < 0.001	*P* = 0.303
Model 5						
Parents alcohol problem						
No (referent)	1.0	1.0	1.0	1.0	1.0	1.0
Yes	1.36 (0.87–2.11)	2.04 (1.03–4.03)	2.12 (1.03–4.36)	1.32 (0.67–2.60)	1.50 (1.00–2.28)	0.87 (0.43–1.75)
Parental death						
No (referent)	1.0	1.0	1.0	1.0	1.0	1.0
Yes	1.02 (0.70–1.49)	0.72 (0.32–1.60)	1.13 (0.52–2.44)	1.10 (0.60–1.99)	1.05 (0.74–1.48)	0.64 (0.34–1.23)
Parent's divorce						
No (referent)	1.0	1.0	1.0	1.0	1.0	1.0
Yes	1.16 (0.37–3.65)	0.87 (0.11–6.92)	0.70 (0.08–6.11)	0.70 (0.15–3.54)	1.30 (0.47–3.59)	2.57 (0.77–8.54)
Poor parenting						
No (referent)	1.0	1.0	1.0	1.0	1.0	1.0
Yes	1.36 (0.99–1.88)	1.28 (0.72–2.28)	1.62 (0.89–2.95)	1.03 (0.60–1.77)	1.51 (1.12–2.03)	1.11 (0.68–1.80)
Quarrelsome home						
No (referent)	1.0	1.0	1.0	1.0	1.0	1.0
Yes	1.99 (1.29–3.07)	1.26 (0.54–2.93)	2.61 (1.20–5.69)	1.66 (0.83–3.34)	1.88 (1.24–2.85)	1.28 (0.65–2.52)
Unhappy childhood						
No (referent)	1.0	1.0	1.0	1.0	1.0	1.0
Yes	1.80 (1.17–2.77)	1.51 (0.64–3.61)	1.40 (0.53–3.72)	0.84 (0.36–1.95)	1.60 (1.05–2.43)	1.27 (0.62–2.58)
ACE score						
0 (referent)	1.0	1.0	1.0	1.0	1.0	1.0
1	1.36 (0.85–2.18)	2.74 (0.95–7.93)	2.30 (0.77–6.93)	1.61 (0.74–3.49)	1.64 (1.05–2.55)	1.02 (0.55–1.92)
2	1.18 (0.71–1.98)	1.48 (0.46–4.78)	1.93 (0.59–6.29)	1.66 (0.73–3.78)	1.49 (0.92–2.41)	0.65 (0.31–1.33)
3	2.57 (1.49–4.43)	3.62 (1.12–11.73)	5.98 (1.83–19.50)	1.83 (0.71–4.71)	3.14 (1.88–5.26)	1.35 (0.62–2.92)
	*P* = 0.002	*P* = 0.077	*P* = 0.005	*P* = 0.212	*P* < 0.001	*P* = 0.721

*Test for trend for ACE score categories (0, 1, 2, 3).

Model 1: adjusted for age, examination year.

Model 2: the same as model 1 and childhood socioeconomic position, education.

Model 3: the same as model 1 and adulthood socioeconomic position (income level, occupation).

Model 4: the same as model 1 and behavioural factors in adulthood (body mass index, leisure time physical activity, smoking, alcohol consumption).

Model 5: the same as model 2 and adulthood socioeconomic position, and behavioural factors in adulthood.

## Appendix

### A. Childhood Questions from the Kuopio Ischaemic Heart Disease Risk Factor Study (KIHD) Baseline Questionnaire

(1) What kind of person was your father when you were a child?

**Table:**

Gentle	0	1	2	3	4	5	Strict/stern
Teetotaler	0	1	2	3	4	5	Drank lot of alcohol

(2) What kind of person was your mother when you were a child?

**Table:**

Gentle	0	1	2	3	4	5	Strict/stern
Teetotaler	0	1	2	3	4	5	Drank lot of alcohol

(3) Changes in your childhood when you were under 10 years old.

**Table:**

	No	yes
Father died	0	1
Mother died	0	1
Parents separated or divorced	0	1

(4) What was your childhood home like when you were about 10 years old?

**Table:**

Quarrelsome	0	1	2	3	4	Peaceful

(5) Was your childhood (when you were under 10 years old)?

**Table:**

0	Easy, happy and safe
1	Ordinary
2	Unhappy and difficult
